# Gastrointestinal Involvement of Testicular Germ Cell Tumor: A Case Report and Literature Review

**DOI:** 10.1155/2017/4789259

**Published:** 2017-10-10

**Authors:** Oluwaseun Shogbesan, Abdullateef Abdulkareem, Asad Jehangir, Sunila Byreddy, Sharon Swierczynski, Anthony Donato

**Affiliations:** ^1^Department of Internal Medicine, Reading Health System, Sixth Avenue and Spruce Street, West Reading, PA 19611, USA; ^2^Hospitalist Services, Reading Health System, Sixth Avenue and Spruce Street, West Reading, PA 19611, USA; ^3^Department of Pathology, Reading Health System, Sixth Avenue and Spruce Street, West Reading, PA 19611, USA

## Abstract

Testicular germ cell tumors (GCT) are the commonest solid tumors in young men. Typical presentation is with painless scrotal swelling; however, symptoms related to complications or metastasis may be the initial presentation. Gastrointestinal (GI) metastasis is seen in about 5% of patients with germ cell tumors and presentation is commonly with GI bleed. It is important to have GCT as a differential diagnosis of GI bleed in young men presenting with unexplained anemia as direct questioning about scrotal swelling and genital examination when appropriate will guide further investigation and facilitate prompt diagnosis. We present a case of a 26-year-old man with testicular germ cell tumor and severe anemia secondary to extension and perforation of duodenum by retroperitoneal metastasis and a review of the literature on the gastrointestinal manifestations of testicular germ cell tumors.

## 1. Introduction

Testicular germ cell tumors (GCT) make up 95% of testicular cancers and are the commonest solid malignancies in young men [1]. Classic presentation is as a painless testicular swelling; however, in 10% of patients, presentation is variable and related to site of metastasis and complications. Gastrointestinal (GI) metastasis is seen in 5% of germ cell tumors and rare presentation with hemodynamically significant gastrointestinal bleed or as occult bleed have been reported [2, 3].

## 2. Case Report

A 26-year-old previously healthy man presented to the emergency department (ED) after an outpatient computed tomography (CT) of the chest showed large burden pulmonary embolus and a 13 mm left lower lobe nodule. He had previously presented at his primary care physician's office with complaints of low back pain, shortness of breath, chest pain, and bilateral leg swelling that had prompted the imaging. The patient reported severe low back pain for 2 weeks with limitation of movement due to pain prior to onset of shortness of breath, chest pain, and bilateral leg swelling. There was no associated cough or hemoptysis. History was otherwise unremarkable including no prior history of personal or family history of cancers or thrombotic conditions, recent surgery, or trauma. He had a 6-pack-year history of tobacco use.

On presentation in the ED, vital signs were significant for tachycardia (pulse rate of 107 beats/minutes), blood pressure of 121/62 mmHg, temperature of 36.9°C, and 99% oxygen saturation on room air.

Physical examination revealed a pale patient with bilateral lower extremity edema without tenderness or erythema. Other examinations including abdominal examination were unremarkable; testicular examination was not documented.

Laboratory data showed significant anemia with hemoglobin of 5.6 g/dl (normal: 14.0–17.5 g/dl; last known hemoglobin from 9 years earlier was 15.7 g/dl), white blood count of 6200/*µ*L (normal: 4800–10800/*µ*L), platelet count 264,000/*µ*L (normal: 130,000–400,000 *µ*L), mean corpuscular volume 82.7 fL (normal: 80.0–99.9 fL), and mean corpuscular hemoglobin 27.5 pg (normal: 27–34 pg). Iron studies were consistent with iron deficiency anemia with ferritin of 18 ng/ml (normal: 27–300 ng/ml), iron of <10 mcg/dL (normal: 50–212 mcg/dL), iron saturation < 3% (normal: 20–50%), and high normal TIBC of 390 ug/dL (normal: 250–425 ug/dL). Electrolytes and hepatic function were within normal limits. Troponin was negative and BNP was 27 pg/ml (0–100 pg/ml). Patient admitted to taking Ibuprofen 400 mg twice a day for back pain for 2 weeks but denied hematochezia, melena, or bleeding from any site. He declined a digital rectal examination.

**Figure 1:**
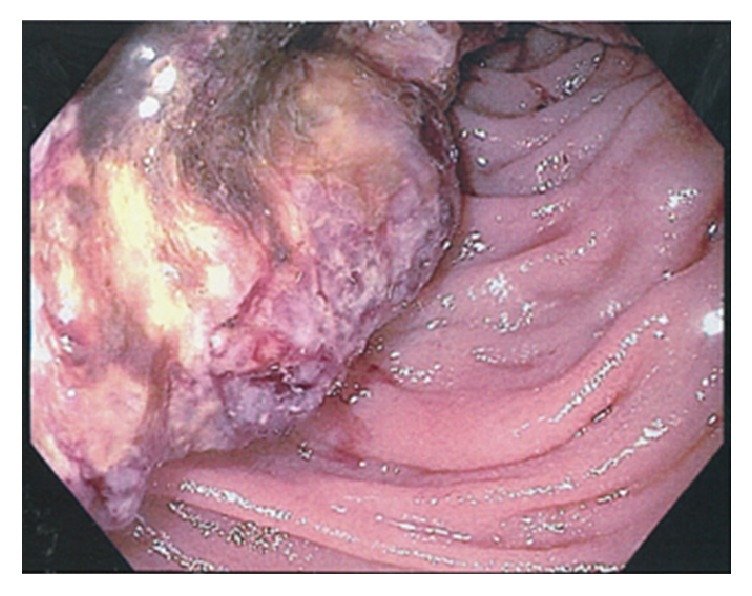
Ulcerated friable duodenal mass noted on esophagogastroduodenoscopy.

Esophagogastroduodenoscopy (EGD) to further investigate his anemia revealed an ulcerated, friable mass within the mid second to third portion of the duodenum (Figure 1). Multiple biopsies were taken with a recommendation for further work-up for duodenal mass.

A CT scan of abdomen and pelvis found a large soft tissue density mass-like structure in the right retroperitoneal space measuring up to 13.4 cm × 9.2 cm extending into the second and third portion of the duodenum with a contained perforation of the duodenum. In addition, a 3.4 × 2.7 cm enlarged para-aortic lymph node and thrombosis of the IVC extending to the right renal vein was also noted. Magnetic resonance imaging of the brain was normal.

Given findings on EGD and CT scan, additional history was obtained which revealed a history of testicular trauma many years priorly when he was hit by a baseball in the right testicle with persistent scrotal swelling; however, the swelling had progressively worsened in the past 1 year. Testicular exam showed a firm right scrotal swelling, twice the size of the left testicle. Scrotal ultrasound revealed 4 separate masses. B-HCG was elevated to 1468 mIU/mL (normal 0–4 mIU/mL), LDH was 367 IU/L (normal 140–271 IU/L), and alpha fetoprotein (AFP) was normal (0.9 ng/mL).

**Figure 2:**
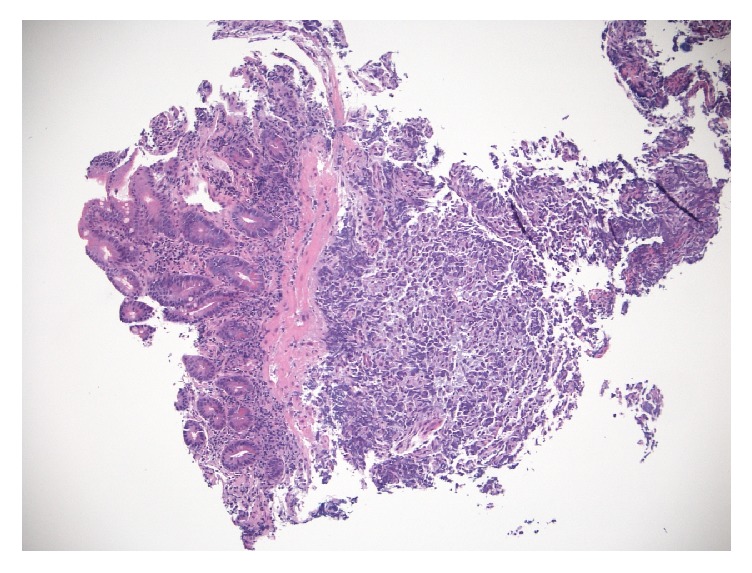
Duodenal mass, metastatic seminoma (H&E stain, magnification ×40).

**Figure 3:**
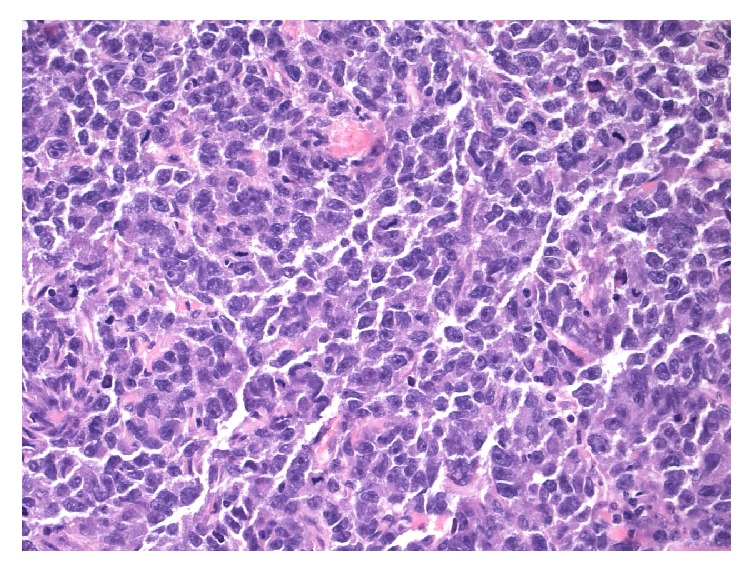
Duodenal mass, metastatic seminoma (H&E stain, magnification ×400).

**Figure 4:**
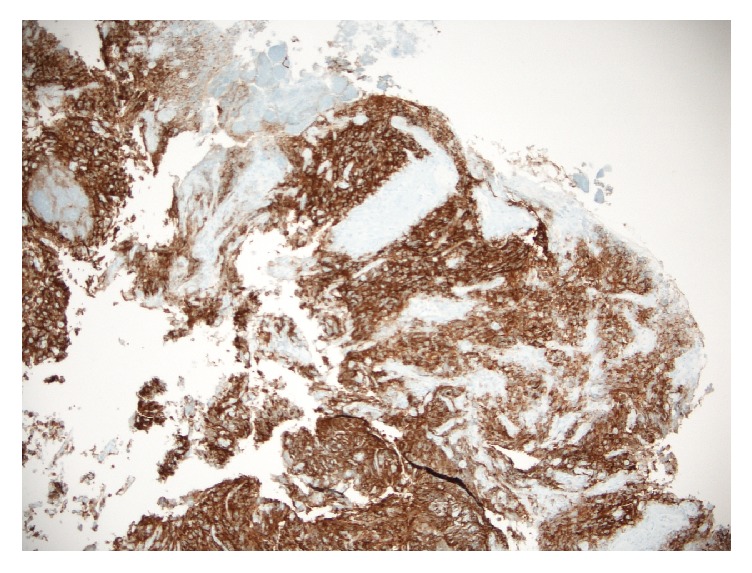
Duodenal mass with metastatic seminoma (PLAP IHC stain, magnification ×200).

Histopathology of the duodenal mass showed a poorly differentiated malignant neoplasm strongly positive for placenta alkaline phosphatase (PLAP), consistent with a malignant germ cell tumor (Figures 2–4).

**Figure 5:**
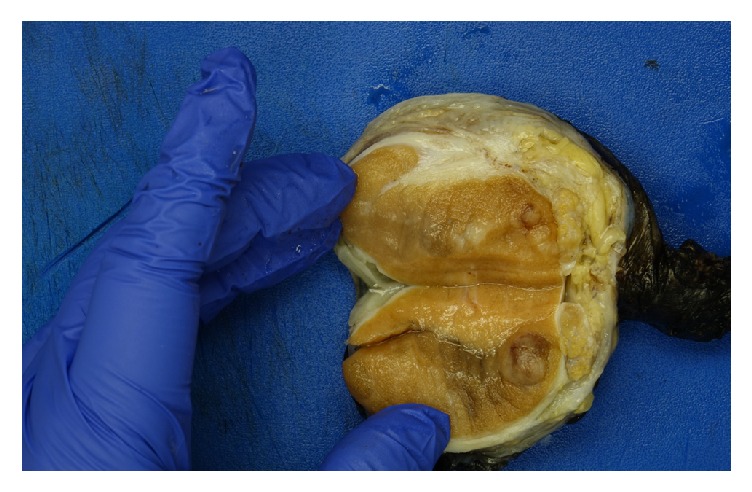
Gross right testis, bisected, showing nodules within the testis.

He underwent a right radical orchiectomy. Gross examination of the right testis revealed four distinct tumor nodules in the testis (Figure 5).

**Figure 6:**
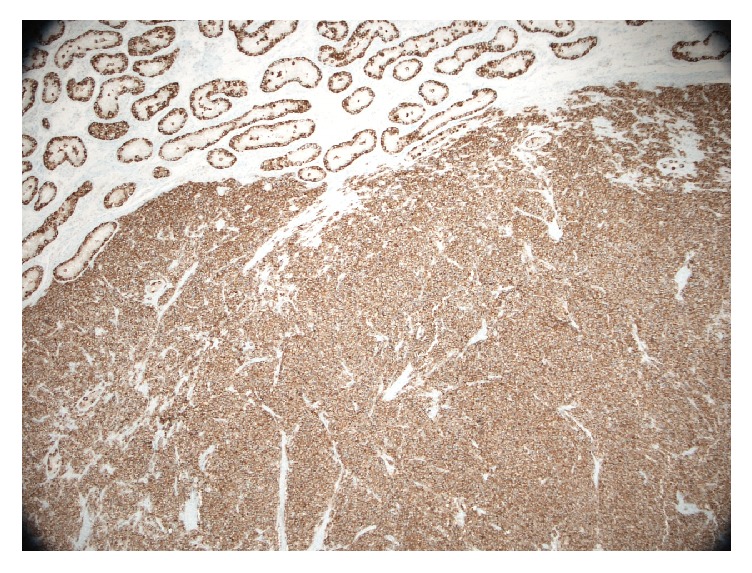
Testicular seminoma (PLAP IHC stain, magnification ×40).

Two of the nodules showed reactivity for beta-HCG. Sporadic CK7 immunoreactivity was seen in the nodules expressing beta-HCG. All four nodules showed immunoreactivity for PLAP (Figure 6).

The pathologic findings were consistent with a seminoma. He was staged as Stage IIIA, pT1N3M1aS1 given the metastases to the retroperitoneum and lungs.

Lower extremity Doppler was negative for DVT. Given burden of blood clot and severe anemia with occult gastrointestinal bleed, an inferior vena cava (IVC) filter was urgently placed. During IVC placement, occlusion of the infrarenal IVC with filling defects extending into the right renal vein was noted. He was subsequently started on anticoagulant given extensive clot burden and underlying malignancy.

He initially received standard chemotherapy with bleomycin, etoposide, and cisplatin, but bleomycin was discontinued after the first cycle when surveillance PFT showed diffusion abnormalities. Patient completed 4 cycles of chemotherapy (etoposide and cisplatin). Imaging following completion of chemotherapy showed complete resolution of lung nodule but greater than 3 cm residual retroperitoneal mass with undetectable HCG, but LDH remained elevated at 314 IU/L. Patient is scheduled for a 6-week follow-up PET scan per NCCN clinical guidelines.

## 3. Discussion

Our patient presented primarily with shortness of breath related to pulmonary embolism and severe anemia. Additionally, extensive thrombosis with bilateral pulmonary embolism, inferior vena cava (IVC), and right renal vein thrombosis were also found. This patient's symptoms related to metastasis included debilitating back pain from retroperitoneal metastasis with extension and perforation of the duodenum, likely resulting in gastrointestinal bleeding and severe anemia. Interestingly, our patient did not report hematochezia or melena.

GI manifestation of GCT is uncommon (5%) with duodenal involvement seen in 1.4% of cases [2]. Gastrointestinal bleeding from site of metastasis following chemotherapy has also been reported [4–6]. Mechanisms include GI perforation following chemotherapy-induced tumor necrosis and shrinking of metastatic gastrointestinal tumor following chemotherapy [7]. Other chemotherapy-related complications involving the GI tract include cases of neutropenic colitis following salvage therapy for refractory nonseminomatous GCT of the testis [8, 9].

There is also an increased risk of stomach cancer following radiotherapy among survivors [10–12]. Overall, gastrointestinal second cancers were higher in seminomas compared to nonseminomas in a review of 5533 survivors in Swedish cancer database [13]. Gastrointestinal symptoms can also be the first symptom of recurrence in patients with previously treated GCT. GCT presenting as abdominal pain with finding of polypoid masses in the gastrointestinal tract within 2 years of orchiectomy and chemotherapy have been reported [14, 15].

Because gastrointestinal presentations of GCT are unusual, they are often not suspected on presentation. A complete physical examination including a scrotal exam may provide the initial clues as to the potential etiology and should not be overlooked, especially since testicular cancers are generally quite curable [16]. Of the estimated 8720 new testicular cancer diagnoses of 2016, less than 400 deaths were anticipated [1]. However, correctly classifying patient and accurate diagnosis play a vital role in achieving good outcomes.

There have been reports of “burnt-out” testicular cancer with presentation related to sites of metastasis in the absence of testicular findings on physical examination or ultrasound. Stokes and Perkins reported a 22-year-old male with metastatic testicular choriocarcinoma with metastasis to the stomach, lungs, liver, kidney, and brain, however, with grossly normal testis with autopsy findings of foci area of scarring and calcification within the testis [17]. Similarly, Altamar et al. reported a 20-year-old with metastatic testicular seminoma with pure seminoma of right testis and concurrent embryonal carcinoma in duodenal mass. A finding of 0.6 cm focus of scarring with calcification likely representing burnt-out primary embryonal tumor versus differentiation at the site of metastasis was a possible explanation [18]. Balogun-Ojuri et al. reported a case of possible primary seminoma of the small bowel with mesenteric nodal involvement. A primary small bowel seminoma was made given normal AFP, normal HCG, and ultrasonographically normal testis. The patient however had a history of undescended testis with orchidopexy and the possibility of jejunal metastasis from an occult primary testicular lesion was entertained [19]. These findings are important as these patients are more likely to present with metastatic symptoms including GI symptoms than testicular mass or swelling.

In a review of patients with GCT with GI symptoms at first presentation, we found 24 publications totaling 25 patients (see Supplementary Table 1 in Supplementary Material available online at https://doi.org/10.1155/2017/4789259). Mean age was 30.44 years. Sixty percent of patients had NSGCT with the number increasing to 80% when combined with mixed GCT which is treated similarly to NSGCT (Table 1). Prior reviews have demonstrated that NSGCT are more likely to have GI involvement [20, 21]. The commonest GI manifestations were abdominal pain (46%) and melena (44%). Other presentation of GI bleeding including hematemesis and hematochezia accounted for 24% of initial presentation. Mean hemoglobin was 6.8 g/dl with patients requiring large volume transfusion to maintain hemodynamic stability. Other GI presentations including nonbloody vomiting, abdominal mass, and distension were less common. Testicular symptoms as an initial presenting symptom was much less and seen in only 24% of patients.

Six of the 21 patients with follow-up information died, with a mean time to death of 25 days. Four of deaths were in patients diagnosed with choriocarcinoma, a more aggressive GCT associated with extensive metastasis and varied complication including massive hemorrhage [5, 22].

In conclusion, testicular GCT is a malignancy of the young. Young men presenting with evidence of GI bleed with suggestions of underlying malignancy may not offer information on testicular swelling, even if present. A review of systems that includes scrotal/testicular swelling should be conducted and a genital examination done if appropriate.

## Supplementary Material

Supplemental Table 1: Previously published reports of testicular germ cell tumors presenting de novo (prior to chemotherapy) with gastrointestinal involvement.

## Figures and Tables

**Table 1:** Summary table showing studies reporting gastrointestinal involvement of testicular germ cell tumor.

*Total N*	*25 patients*
Mean age	30.44 years
Range age	16–56 years

	*N (%)*

*Type of GCT*	
Seminoma	5 (20%)
NSGCT	15 (60%)
Mixed	5 (20%)

	*N (%)*

*Range of GI symptoms*	
Melena	11/25 (44%)
Abdominal pain	9/25 (36%)
Abdominal mass	4/25 (16%)
Hematochezia	2/25 (8%)
Hematemesis	4/25 (16%)
Vomiting, nonbloody	3/25 (12%)
Others (abdominal distension, rigid abdomen)	2/25 (8%)
Mean hemoglobin at presentation (g/dl)	6.8
*EGD done (%)*	21/24 (84%)

	*N*

*Laterality of testis involved*	
Right	14
Left	5
Diffuse scrotal swelling	1
NA	3
Normal testis on Ultrasound	2
*Testicular symptoms (swelling or pain) as initial presenting complaint (%)*	6/25 (24%)

*Outcome*	

Survived *N* (%)	Mean follow up (months) 13.2 Survived 15/21 (71%)
Deaths *N* (%)	Mean days to death 25 Death 6/21 (28%)
Choriocarcinoma	4/6 (66%)
* * Seminoma	1/6 (17%)
Mixed GCT	1/6 (17%)

GCT: germ cell tumor; NSGCT: non seminomatous germ cell tumor; GI: gastrointestinal; EGD: esophagogastroduodenoscopy.
