# Supplementary material for: Gastrointestinal Involvement of Testicular Germ Cell Tumor: A Case Report and Literature Review
Source: Case Rep Gastrointest Med. 2017 Oct 10;2017:4789259. doi: 10.1155/2017/4789259 (PMC5654329; doi:10.1155/2017/4789259)
Supplement: Supplementary file 1 — Supplemental Table 1: Previously published reports of testicular germ cell tumors presenting de novo (prior to chemotherapy) with gastrointestinal involvement. [file 4789259.f1.docx]

Supplemental Table 1. Table showing articles included in review

| **Author Year** | **Type of GCT** | **Age** | **GI symptoms** | **EGD/laparotomy finding** | **Hb (g/dl)** | **Testis involved** | **Outcome** |
| --- | --- | --- | --- | --- | --- | --- | --- |
| Leslie 2003 [23] | Seminoma | 30 | Melena | Duodenal mass with bleed | 9.5 | Right | Survived.  4 months follow-up |
| Rodriguez-Lopez 2015 [24] | Seminoma | 30 | Vomiting, Melena | Partial duodenal stenosis with malignant appearing component | 5.3 | Right | Survived.  12 months follow-up |
| Koksal 2013  [25] | Mixed-90% seminoma, 10% mature teratoma | 32 | Melena | Submucosal duodenal mass with active duodenal bleed | 7.1 | Right | Death.  3 days after first chemo |
| Stokes 1988 [17] | Choriocarcinoma | 22 | Heme occult positive stools | Ulcerated sessile lesion with an overlying clot on greater curvature of stomach. | 7.7 | Grossly normal testis.  Left testis with focal area of scarring and calcification | Death.  1 month after diagnosis. |
| Altamar 2004 [18] | Seminoma | 20 | Bright red blood per rectum.  Tender mass on abdominal exam. | 5 cm friable ulcer and mass in 2nd part of duodenum | 5.4 | Right | NA |
| Komori 2016 [5] | Choriocarcinoma | 56 | Abdominal distension.  Hemoperitoneum after percutaneous needle biopsy | Extragonadal GCT causing massive bleeding and GI hemorrhage | NA | NA | Death.  1 month post op |
| Infante 2004 [26] | Choriocarcinoma | 37 | Melena | 3cm submucosal mass with central ulceration in upper body of stomach.  (colonoscopy)  2 polypoid masses in right colon | 7.7 | Normal testicular ultrasound | Death.  3 weeks post admission |
| Syrigos 2002 [27] | NSGCT (embryonal carcinoma) | 25 | Epigastric pain, melena | Ulcerating infiltrating mass in 2nd portion of duodenum causing narrowing of the lumen | 8.1 | Right | Survived.  24 months follow up |
| Thompson 2004 [28] | Embryonal carcinoma | 26 | Nausea and dark emesis.  Hem positive stools | Large fungating mass like structure in 3rd portion of duodenum. Area around mass was ulcerated | 6.3 | Right | Survived.  1 year follow up |
| Bain 2010 [29] | Choriocarcinoma | 17 | hematemesis | Large nearly obstruction mass in medial wall of 2nd and 3rd portion of duodenum, actively bleeding. Ampulla completely obscured by tumor. | 7.5 | NA | NA |
| Miocinovic 2008 [30] | Seminoma | 45 | Rigid abdomen | Duodenal perforation from paraaortic LN invading 2nd and 3rd portion of duodenum | NA | Right | Death.  4 weeks |
| Krakovska 2008 [31] (Case 1) | Seminoma + teratoma ( mixed GCT) | 31 | None | Fragile, bleeding solid tumor of proximal jejunum with an exulceration | 6.8 | Right | Survived.  Follow-up NA |
| Shibuya 2008 [32] | Choriocarcinoma | 27 | Abdominal pain, melena, vomiting | Red, polypoid lesion with areas of hemorrhage in ant wall of gastric body | 6.5 | Normal Ultrasound | Survived.  2 years follow up |
| Mazumdar 2016 [33] | Mixed GCT | 49 | Abdominal  pain | 1 x 2 cm area of nodularity in post wall of stomach and circumferential growth in second portion of duodenum with luminal stenosis | NA | Right | NA |
| Moore 2009 [34] | Mixed NSGCT (mature teratoma, embryonal carcinoma and choriocarcinoma) | 16 | Abdominal pain | Retroperitoneal mass adherent to the jejunum with hemoperotoneum.  No jejunal erosion or invasion | NA | Left | Survived.  34 months after diagnosis |
| Opdam 1995 [35] | NSGCT | 41 | melena | Multiple gastric polyp and ulcerated proliferative mass in 2nd part of duodenum | 6.6 | Right | Survived. NA |
| Hofflander 1999 [36] | Mixed GCT | 27 | Abdominal pain, melena | Normal EGD & Colonoscopy.  Ex. lap with large lesion in jejunum with mucosal erosion and bleeding  causing intussusception of small bowel | 8 | Left | Survived. NA |
| Gadodia 2011[4] | 1.Choriocarcinoma  2. Mixed NSGCT  (Embroyonal and yolk sac) | 25  21 | 1. 5 x 5 cm lump in right hypochondrium  2. 10 x 10 cm mass in epigastrium | 1.Large friable growth infiltrating the second part of duodenum with active bleed  2. large friable growth with multiple clots in second part of duodenum | NA | Right | Survived |
| Ani 2016 [37] | Seminoma | 30 | Hematemesis, upper abdominal pain | Fungating ulcerated mass invading the second part of duodenum | NA | Left | Survived. 1 year follow up |
| Malhotra 1999 [38] | NSGCT | 17 | Epigastric pain, hematemesis and melena | Large extrinsic impression on the postero-medial wall of the second part of the duodenum with an ulcer at the summit with adherent clot | 4 g/dl | Right | Survived. 3 months follow up |
| Shariat 2005 [39] | NCGCT (Choriocarcinoma) | 51 | hematochezia | Hard fungating mass 30 cm from ileocecal valve.  Five erythematous nodules with overlying ulcer and exudates in jejunum, erythematous nodules along greater curvature of stomach | 5.3 | Right | Death. 41 days after diagnosis. |
| Kamel 2015 [40] | NSCGT (Choriocarcinoma) | 31 | Non bloody non bilious vomiting.  Melena | Duodenitis on EGD.  Capsule endoscopy with actively bleeding lesion in distal small intestine. | 7 | Left | Survived. 2 months follow up |
| Kadakia 1992 [14] | NSGCT | 33 | Epigastric pain | 4-5 cm polypoid lesion in the proximal stomach and a small sessile polypoid lesion in the distal esophagus | NA | NA | NA |
| Laiyemo 2009 [15] | Mixed GCT | 22 | Melena, abdominal pain | 2 cm ulcerated polypoid mass in 2nd part of the duodenum,  1.5 cm smooth polypoid mass lesion in the third portion of duodenum. Biopsy confirmed | 7.6 | Edematous scrotum | Survived. 5 months follow up |

EGD=Esophagogastroduodenoscopy; GI=Gastrointestinal; Hb=Hemoglobin; LN=lymphadenopathy; NA=Not available; NSGCT=Nonseminomatous Germ Cell Tumor;
